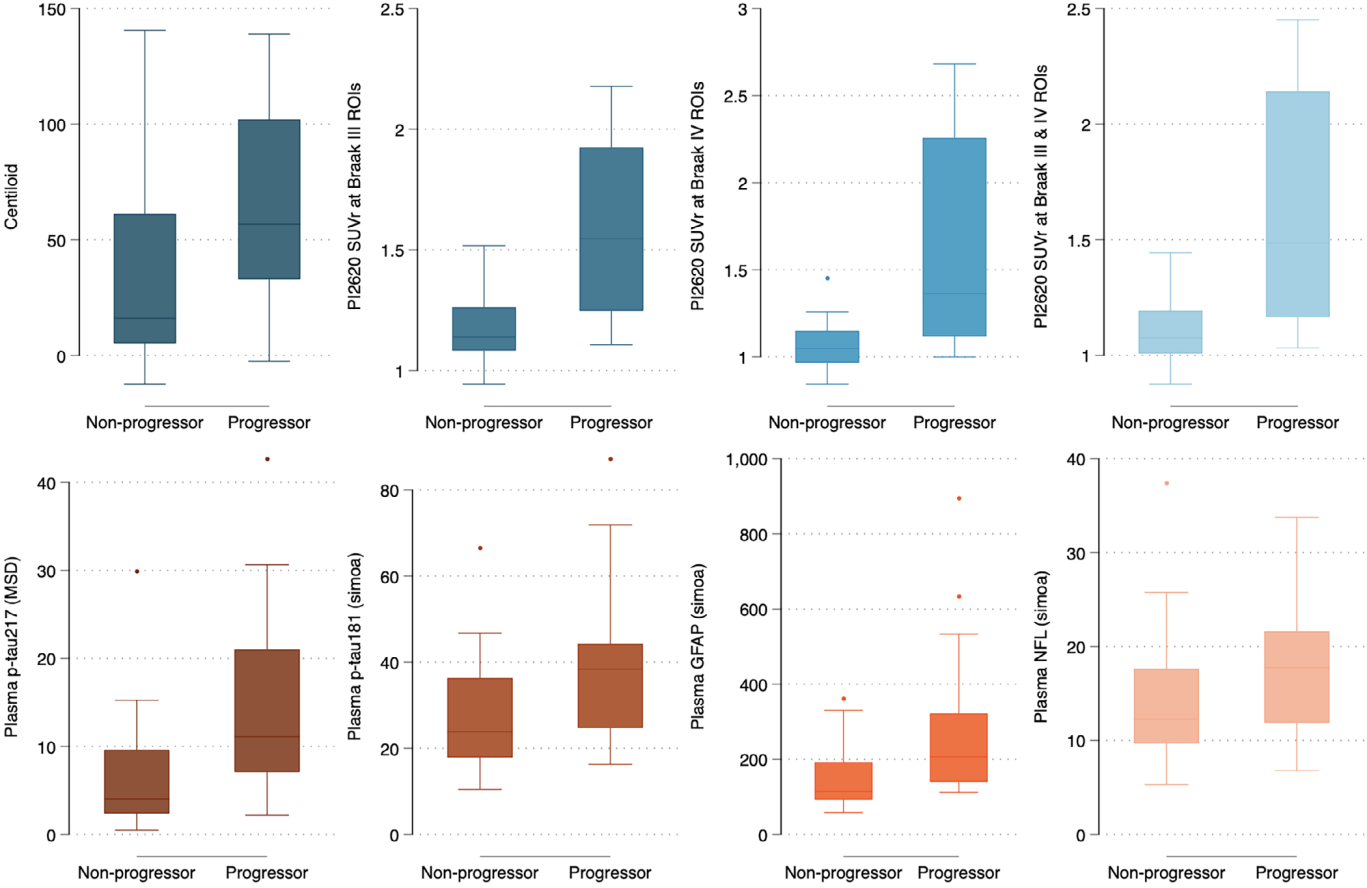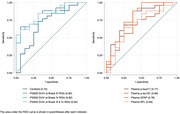# PET and Blood Biomarkers as Predictors of Short‐Term Progression in MCI and Mild Dementia: Prospective Insights from a Southeast Asian Middle‐Income Country

**DOI:** 10.1002/alz70856_100227

**Published:** 2025-12-25

**Authors:** Kittithatch Booncharoen, Akarin Hiransuthikul, Yuttachai Likitjaroen, Kammant Phanthumchinda, Sekh Thanprasertsuk, Poosanu Thanapornsangsuth

**Affiliations:** ^1^ Neurology Center, Phyathai 1 Hospital, Bangkok, Rachathewi, Thailand; ^2^ Neurocognitive Unit, Division of Neurology, Department of Medicine, Faculty of Medicine, Chulalongkorn University, Bangkok, Thailand; ^3^ Memory Clinic, King Chulalongkorn Memorial Hospital, The Thai Red Cross Society, Bangkok, Thailand; ^4^ Department of Preventive and Social Medicine, Faculty of Medicine, Chulalongkorn University, Bangkok, Thailand; ^5^ Department of Physiology, Faculty of Medicine, Chulalongkorn University, Bangkok, Thailand; ^6^ Cognitive, Clinical and Computational Neuroscience (CCCN) Center of Excellence, Chulalongkorn University, Bangkok, Thailand; ^7^ Thai Red Cross Emerging Infectious Diseases Health Science Centre, World Health Organization Collaborating Centre for Research and Training on Viral Zoonoses, King Chulalongkorn Memorial Hospital, The Thai Red Cross Society, Bangkok, Thailand

## Abstract

**Background:**

Alzheimer's disease (AD) specific biomarkers, including blood *p*‐Tau, tau PET, and amyloid PET, are excellent predictors of AD pathology. However, the potential of these biomarkers–along with neuroinflammation markers such as GFAP and NfL–to predict short‐term cognitive and functional decline in patients with mild cognitive impairment (MCI) and mild dementia has not been extensively explored.

**Method:**

Participants with memory‐predominant MCI and mild dementia were recruited from a tertiary care memory clinic in Bangkok, Thailand. At baseline, Tau PET (PI‐2620) and amyloid PET (Florbetaben) were performed, blood samples were collected to measure plasma *p*‐Tau‐181, *p*‐Tau‐217, GFAP, and NfL. Cognitive and functional abilities were assessed using the Thai version of the MoCA and CDR‐SB scales at baseline and a 24‐month follow‐up. “Progressors” were defined as participants who experienced a decline in MoCA scores by >3 points or an increase in CDR‐SB scores by >1 point. We employed Wilcoxon rank‐sum tests to compare biomarker levels between progressors and non‐progressors, and biomarker performance was assessed via ROC analysis.

**Result:**

Among 42 participants (median age: 71 years, 66.7% female), 26 (61.9%) were classified as progressors. Median (IQR) amyloid PET centiloid levels (56.7 [33.0–102.0] vs. 16.1 [5.3–61.3], *p* = 0.026) and Tau PET SUVR for Braak stages 3‐4 (1.5 [1.2–2.1] vs. 1.1 [1.0–1.2], *p* <0.001) were significantly higher in progressors. Plasma *p*‐Tau‐217 (11.1 [7.1–21.0] vs. 4.0 [2.4–9.6] pg/mL, *p* = 0.004) and GFAP levels (206.7 [140.6–322.0] vs. 114.5 pg/mL [92.6–192.7], *p* = 0.003) were also significantly higher in progressors, while plasma *p*‐Tau‐181 and NfL levels showed no significant differences between groups (Figure 1). Tau PET SUVR provided the best prognostic value for 24‐month progression (AUC 0.84, 95% CI 0.71–0.96), followed by plasma GFAP (AUC 0.78, 95% CI 0.62–0.94) and plasma *p*‐Tau‐217 (AUC 0.77, 95% CI 0.61–0.92) (Figure 2).

**Conclusion:**

Tau PET SUVR in Braak stages 3‐4 is a robust marker for short‐term cognitive and/or functional deterioration. Plasma *p*‐Tau‐217 not only serves a diagnosis but also has prognostic capabilities. Plasma GFAP outperforms plasma NfL in predicting short‐term progression. Future studies should focus on the value of these biomarkers in long‐term progression.